# Deep Learning-Based Text Emotion Analysis for Legal Anomie

**DOI:** 10.3389/fpsyg.2022.909157

**Published:** 2022-06-17

**Authors:** Botong She

**Affiliations:** School of Drug Control and Public Security, Criminal Investigation Police University of China, Shenyang, China

**Keywords:** legal anomie analysis, text emotion analysis, deep learning, convolutional neural network, Bi-directional long short-term memory, emotion analysis

## Abstract

Text emotion analysis is an effective way for analyzing the emotion of the subjects’ anomie behaviors. This paper proposes a text emotion analysis framework (called BCDF) based on word embedding and splicing. Bi-direction Convolutional Word Embedding Classification Framework (BCDF) can express the word vector in the text and embed the part of speech tagging information as a feature of sentence representation. In addition, an emotional parallel learning mechanism is proposed, which uses the temporal information of the parallel structure calculated by Bi-LSTM to update the storage information through the gating mechanism. The convolutional layer can better extract certain components of sentences (such as adjectives, adverbs, nouns, etc.), which play a more significant role in the expression of emotion. To take advantage of convolution, a Convolutional Long Short-Term Memory (ConvLSTM) network is designed to further improve the classification results. Experimental results show that compared with traditional LSTM model, the proposed text emotion analysis model has increased 3.3 and 10.9% F1 score on psychological and news text datasets, respectively. The proposed CBDM model based on Bi-LSTM and ConvLSTM has great value in practical applications of anomie behavior analysis.

## Introduction

Anomie behavior refers to a disordered social phenomenon caused by the anomie state of the current law in the process of the transformation of a country’s new and old systems (Liu et al., 2021). Anomie is a phenomenon caused by the disintegration or transformation of social structure. The disintegration of the social structure. Anomie and deviant behavior increased. Under the market economy, the interweaving of various contradictions, the friction of various phenomena and the confrontation of various behaviors have led to more and more “Anomies” and aroused people’s general concern. It has become the main topic of current behavioral law research to think about anomie behavior and seek countermeasures (Fukuda et al., 2021). Teenagers are prone to behavior deviation due to the influence of various factors such as psychology, physiology and living environment (Fukuda et al., 2021). If they are not dredged in time, they will further lead to more serious anomie behavior. Social work adheres to the concept of helping others and self-help and should actively intervene in teenagers’ anomie behavior. In recent years, cases of juvenile anomie have occurred frequently, and its social harm is also serious. Therefore, it is the responsibility of adolescent social workers to help anomie adolescents return to the normal life and solve the potential safety hazards in society (Angioletti et al., 2022).

As a computerized text analysis method, deep learning model is currently applied by researchers in many studies in the field of psychological text analysis (Phan and Rauthmann, 2021). In the research field of psychological counseling, the deep learning model can be used to explore the topic of conversation between Counselors and parties in the counseling process, compare the similarity of different treatment categories, and code behavior. In social media and mental health, the deep learning-based classification model can be used to identify and predict various psychological disorders and calculate personality. We will pay attention to the improvement of the deep learning algorithms and apply it to explore the psychological connotation in journalistic text.

Text emotion analysis is a common application of the natural language processing (NLP) methods (Ozawa, 2021). It is to identify the emotional polarity (positive or negative or neutral) or emotional intensity of a given text or sentence segment. Emotional analysis is mostly used in product review analysis and public opinion statistical monitoring, which is helpful to business decision support and public opinion guidance of organs and units. Previous studies mostly focused on the construction of artificial dictionary and feature extraction. However, the construction of emotional dictionary is time-consuming, laborious, and difficult to maintain. Artificial feature extraction needs expert domain knowledge (Sun et al., 2022). Word vector technology, which has sprung up in recent years, has become the basic technology in natural language processing. However, the popular word vector model is mainly obtained by learning context information. That is, it focuses on semantic information rather than emotional information concerned by emotion analysis tasks. As an improved model of RNN network (Wang et al., 2012), long-term and short-term memory model (LSTM; Wang et al., 2012) can make better use of the long-distance dependence information in sequence data, which is suitable for text emotion classification.

Convolutional neural network (CNN; Tan et al., 2021) is an important feature extraction model for psychological text. Because of its strong local feature extraction ability, it has achieved good results in the field of text classification. However, in the face of the huge amount of data and more categories in some text classification tasks, the traditional CNN model exposes the shortcomings of low calculation efficiency, slow training speed and easy over fitting, which then affects the effect of classification (Sun et al., 2021). Therefore, how to optimize the CNN model structure or improve the model algorithm to effectively solve the problem of large-scale text classification is the focus of deep learning classification model research (Agga et al., 2021). When dealing with short text emotion classification, CNN uses convolution layer to extract local features and maximum pooling layer to select the maximum value of local features, which is easy to ignore the long-term sequence characteristics of the texts. This paper uses a new deep learning model Convolutional Long Short-Term Memory (ConvLSTM; Wang et al., 2021), which uses the long LSTM to replace the maximum pool layer in CNN, to reduce the loss of local information and capture the long-term dependence in sentence sequences.

Previous studies have proved that the fusion of different text features can provide more information for the classifier. However, how to construct the word embedding method and the structure of the classifier and integrate different features to make the proposed model have better classification effect is a focus of this paper. In other words, when judging the emotional polarity of sentences, we should not only consider combining more text word information, but also consider constructing classifier structure to better extract different features in the text.

In this paper, we propose a word embedding, and splicing mechanism based on Bi-LSTM (Li et al., 2021) and ConvLSTM, called Bi-direction Convolutional Word Embedding Classification Framework (BCDF). It can not only express the word vector in the text, but also embed the part of speech tagging information as a feature of sentence representation. In addition, an emotional parallel attention mechanism is proposed, which uses the temporal information of the parallel structure calculated by Bi-LSTM to update the storage unit through the gating mechanism. The convolutional layer can better extract certain components of sentences (such as adjectives, adverbs, nouns, etc.), which play a more significant role in the expression of emotion. On this basis, a ConvLSTM network is designed to further improve the classification results. It can be understood that in a sentence, some words are the key words of the sentence. Here, the ConvLSTM mechanism is to help extract this kind of focus and better show the focus in the feature. Experimental results show that compared with benchmark models, this method has better classification performance in terms of the average accuracy and F1 score. The main contribution of this work is as follows:

(1) Proposes a new word embedding and splicing mechanism BCDF based on Bi-LSTM and ConvLSTM. BCDF can express the word vector in the text, and embed the part of speech tagging information as a feature of sentence representation.
(2) Uses an emotional parallel attention mechanism to calculate the temporal information of the parallel structure to update the storage unit through the gating mechanism.
(3) To take advantage of the strong ability of convolutional layer, this work designs a ConvLSTM network to capture the features of word vectors and to further improve the classification results.
(4) Conducts comprehensive experiments to evaluate the performance of BCDF. The evaluation results indicate that BCDF has the highest F1 for text analysis compared with traditional methods.

The structure of this paper is as follows: Section 2 introduces the related work of news and psychological text analysis and their impact on anomie behaviors. Section 3 introduces the proposed BCDF model for word embedding and emotion classification. Section 4 presents the experiment settings and results. Section 5 concludes this paper.

## Related Work

We analyze the related work from two aspects: the application of machine learning-based text analysis in legal anomie analysis area, and the development of the intelligent text emotion analysis in psychological health analysis area.

### The Application of Machine Learning-Based Text Analysis

Hamilton and Davison (2022) discussed some of the legal and ethical issues that come with machine learning in the text analysis context, as well as some suggestions for managers to use in determining the suitability of machine learning projects. Lai and Tan (2019) utilized deception detection as a testbed to see how we might leverage machine learning models’ explanations and predictions to improve human performance while keeping human agency and show that there is a trade-off between human performance and human agency, and that explanations of machine forecasts can help to mitigate this trade-off. Tung (2019) brought the legal function up to date with today’s omnipresent transformations It also serves as a reminder to business leaders that the legal function, such as corporate legal strategists, will be required to drive and maintain change at the convergence of law, business, and technology. Verma et al. (2020) created a rubric with desirable qualities of counterfactual explanation algorithms to counterfactual explainability. Mehrabi et al. (2021) created a taxonomy for fairness definitions that machine learning researchers have defined to avoid the existing bias in AI systems.

### The Development of the Intelligent Text Emotion Analysis in Psychological Health Analysis Area

Tate et al. (2020) developed a model that can predict mental health problems in mid-adolescence and investigate if machine learning techniques will outperform logistic regression. It may be unnecessary for similar studies to forgo logistic regression in favor of other more complex methods. Ji et al. (2022) analyzed the motivation to avoid unbearable psychological pain, coupled with the decision-making bias of underestimating the value of life, is a high predictor of suicide attempt. The analysis also showed that there were differences in potential mechanisms between suicidal ideation and attempted suicide because suicidal ideation was more related to despair. Mohr et al. (2017) critically reviewed the research on personal perception related to mental health, mainly focusing on smart phones, but also including the research on wearable devices, social media, and computers. Linthicum et al. (2019) introduced machine learning and its potential application to open questions in suicide research. Chancellor et al. (2019) studied how scientific papers represent human research subjects in Human-centered machine learning and discussed the tensions and impacts of interdisciplinary research.

## Bi-Direction Convolutional Word Embedding Classification Framework for Text Emotion Analysis

### Bi-LSTM-Based Word Embedding and Representation

In general, the true meaning of metaphors depends not only on their original meaning, but also on the frontward and backward words. To synthesize the word meaning of the expression words, the word vector of the static sense and text sense is used as the original representation of the input order. The Bi-directional Long Short-Term Memory (Bi-LSTM) model is used to learn the text representation of the journalistic and psychological words. Given an input sentence with N words T = {*a*_1_,*a*_2_,…,*a*_*N*_}. Combining the two types of words to represent the original meaning of words *m*_*i*_, as shown in Eq. 1.


(1) $$m_{i}=\left[f_{i},\ldots ,f_{i+l}\right]$$

where *f*_*i*_ is a static word embedding.

Based on the words *m*_*i*_ (i = 1,2,…,N), we use Bi-LSTM sequential encoder to generate cultural word representation. Physically, LSTM sheet consists of a gate structure, in which the input gate controls the information that is input into the nerve sheet. The legacy gate determines which information is discarded from the nerve sheet. The output gate determines which information is output from the nerve sheet. In addition, the state value records all the useful historical information of the current moment. By using the forward LSTM and the text before the current word in the sentence, the word representation of *x*_*i*_ is calculated by the Eqs. 2–7.


(2) $$x_{i}=\sigma \,\left(W_{z}\,m_{i}+G_{z}\,{\vec{h}}_{i-1}+v_{z}\right)$$

(3) $$o_{i}=\sigma \,\left(W_{o}\,m_{i}+G_{o}\,{\vec{h}}_{i-1}+v_{o}\right)$$

(4) $$f_{i}=\sigma \,\left(W_{f}\,m_{i}+G_{f}\,{\vec{h}}_{i-1}+v_{f}\right)$$

(5) $${\tilde{d}}_{i}=t\,a\,n\,h\,\left(W_{c}\,m_{i}+G_{c}\,{\vec{h}}_{i-1}+v_{c}\right)$$

(6) $$d_{i}=z_{i}⊕{\tilde{d}}_{i}+f_{i}⊕c_{i-1}$$

(7) $${\vec{h}}_{i}=o_{i}⊕\text{tanh}\,\left(d_{i}\right)$$

where, *x*_*i*_, *o*_*i*_, represent the input and the output gates, respectively, *d*_*i*_ is the text memory content, $\,{\tilde{d}}_{i}$ is the new memory content, and ${\vec{h}}_{i}\,$is the hidden output of the forward LSTM. *W*_*z*_, *W*_*o*_, *W*_*f*_ and *W*_*c*_ are the weight parameters of the current input *a*_*i*_, *G*_*z*_, *G*_*o*_, *G*_*f*_ and *G*_*c*_ are the weights of the hidden layer state ${\vec{h}}_{i-1}$, and *v*_*o*_, *v*_*z*_, *v*_*f*_ and *v*_*c*_ are the values of the output gate, input gate, legacy gate and hidden single layer state, respectively. Tanh and σ, respectively, represent the tangent and sigmoid functions.

Using the directional LSTM, the text representation is calculated based on the contextual words of *a*_*i*_ in each sentence, as shown in Eq. 8.


(8) $${\overset{\leftarrow }{j}}_{i}=\overset{\leftarrow }{L\,S\,T\,M}\left(a_{i},{\overset{\leftarrow }{j}}_{i-1},{\overset{\leftarrow }{\theta }}_{i}\right)$$

where ${\overset{\leftarrow }{\theta }}_{i}\,$ is all the parameters of the backward LSTM unit.

Equation 9 summarizes the calculation steps of the forward LSTM:


(9) $${\vec{j}}_{i}=\vec{L\,S\,T\,M}\,\left(a_{i},{\vec{j}}_{i-1},{\vec{\theta }}_{i}\right)$$

where ${\vec{\theta }}_{i}$ is the parameters of the forward LSTM.

### Convolutional Long Short-Term Memory-Based Word Embedding Classification Bi-Direction Convolutional Word Embedding Classification Framework

The proposed classification framework BCDF consists of two stacked ConvLSTM layers, one Flatten layer, one Dropout layer, and one Dense layer. The role of the Dropout layer is to prevent overfitting, and the dropout rate is 0.3. In the last Dense layer, the activation function used is Softmax. The input of BCDF is a word vector containing the contextual information of the middle word, namely ContWord. A ContWord is denoted as $B_{j}=\left\{b_{j}^{1},b_{j}^{2},\cdots ,b_{j}^{L}\right\}$ (L = 400), where j refers to the jth ContWord, and L refers to the number of sampling points contained in a ContWord.

The essence of ConvLSTM is the same as LSTM, which uses the output of the previous layer as the input of the next layer. ConvLSTM adds convolution operations, which is different from the classical LSTM. Therefore, ConvLSTM can obtain the temporal relationship and extract spatial features as a convolutional layer. In ConvLSTM, the switching between states is also replaced by convolution calculations. Equations 10–14 show the state transitions of the ConvLSTM.


(10) $$I_{t}=\sigma \,\left(W_{x}^{I}*X_{t}+W_{h}^{I}*H_{t-1}+W_{c}^{I,{}^{\circ}}\,C_{t-1}+b_{I}\right)$$

(11) $$F_{t}=\sigma \,\left(W_{x}^{F}*X_{t}+W_{h}^{F}*H_{t-1}+W_{c}^{F,{}^{\circ}}\,C_{t-1}+b_{F}\right)$$

(12) $$C_{t}=F_{t}^{{}^{\circ}}\,C_{t-1}+I_{t}^{{}^{\circ}}\,t\,a\,n\,h\,\left(W_{x}^{C}*X_{t}+W_{h}^{C}*H_{t-1}+b_{C}\right)$$

(13) $$O_{t}=\sigma \,\left(W_{x}^{O}*X_{t}+W_{h}^{O}*H_{t-1}+W_{c}^{O,{}^{\circ}}\,C_{t-1}+b_{O}\right)$$

(14) $$H_{t}=O_{t}^{{}^{\circ}}\,t\,a\,n\,h\,\left(C_{t}\right)$$

where, H, W, *, and ∘ represents a hidden state, a filter, the convolution operator, and the Hadamard product, respectively. I_t,χt_, *F*_*t*_, *C*_*t*_, *o*_*t*_ and b denotes an input door, the input, a forgotten door, a cell state, an output door, and a bias, respectively.

In BCDF, the number of cells in each layer is shown in Table 1. The training parameter settings of BCDF is shown in Table 2.

**TABLE 1:** The numbers of cells in different layers.

Layer	The number of cells	Kernel size
1_*st*_ConvLSTM	80	(80,4)
2_*nd*_ConvLSTM	40	(40,4)
Flatten	40	–
Dropout	40	–
Dense	5	Unit(5)

**TABLE 2:** The training parameter settings of BCDF.

Parameter	Setting
Training epoch	30
Loss function	Sparse categorical cross-entropy
Optimizer	Adam
Learning rate	0.001
Dropout rate	0.3

## Experimental Design and Results Analysis

The experimental device is a computer equipped with an NVIDIA GeForce GTX 950M and a GPU with 3049 MB of memory. Two datasets are used to evaluate the proposed BCDF model: the emotional analysis dataset published on Audio/Visual Emotion Challenge and Workshop’19 (AVEC’19; Ringeval et al., 2019) and Google’s GoEmotions dataset (Demszky et al., 2020).

The experiment uses four evaluation metrics to evaluate the classification performance of BCDF. The four metrics are f1 score (F1), recall rate (Rec), precision rate (Pre), and overall accuracy rate (Acc). Formula (6) to Formula (9) show how they are calculated.


(15) $$\mathrm{Pr}e=\frac{t\,p}{t\,p+f\,p}$$

(16) $$R\,\text{ec}=\frac{\text{tp}}{t\,p+f\,n}$$

(17) $$A\,c\,c=\frac{\text{tp}+t\,n}{t\,p+t\,n+f\,p+f\,n}$$

(18) $$F\,1=\frac{2*P\,\text{re}*R\,e\,c}{\mathrm{Pr}e+R\,e\,c}$$

where, tp is true positives, fn is false negatives, tn is true negatives, and fp is false positives.

### Emotional Word Recognition of Different Components of a Sentence

To verify the effectiveness of the proposed BCDF model, it is compared with the state-of-the-art methods based on the two data sets AVEC and GoEmotions. Table 3 shows the results of the emotional word recognition in different components of a sentence.

**TABLE 3:** Emotional word recognition in different components of a sentence (%).

Model	AVEC			GoEmotions		
	Pre	Rec	F1	Pre	Rec	F1
RNN	61.8	71.2	64.3	62.1	67.9	63.2
CNN + RNN	70.4	72.6	71.7	75.6	57.2	62.7
LSTM	70.8	73.3	72.4	68.9	62.2	65.9
CNN + LSTM	72.2	74.7	73.5	68.7	64.5	66.9
BCDF	74.1	77.1	75.6	74.3	68.8	69.0

From Table 3, we can see that RNN performs worse than other models. CNN + RNN is better than RNN alone. The reason may be the CNN + RNN model can extract both the contextual and temporal features from a long sentence. LSTM performs similar as the CNN + RNN model, while CNN + LSTM performs better than LSTM. The proposed BCDF achieves the optimal performance by comparing with the four traditional models. The F1’s of BCDF on Data1 and Data2 are 75.6 and 69%, respectively, which are 1 and 2% higher than those of CNN + LSTM based on Data1 and Data2, respectively. The proposed BCDF model is better than the compared traditional methods in all psychological texts. Compared with classical methods, the BCDF method is easier to identify the journalistic words in news.

### Psychological Word Recognition Evaluation

To evaluate the proposed BCDF model, a psychological word recognition experiment is conducted based on GoEmotions datasets.

We can see from Table 4 that BCDF performs the best on both GoEmotions datasets for recognizing the psychological words. The F1’s of BCDF achieves 81.6 and 88.3%, respectively.

**TABLE 4:** Psychological word recognition results based on datasets GoEmotions (%).

Type	Model	Pre	Rec	F1
AVEC	RNN	67.3	75.2	69.6
	CNN + RNN	72.4	77.5	75.8
	LSTM	71.8	79.4	78.3
	CNN + LSTM	72.8	77.4	75.4
	BCDF	82.1	84.2	81.6
GoEmotions	RNN	66.5	74.8	72.3
	CNN + RNN	76.9	79.8	77.4
	LSTM	72.4	73.3	73.0
	CNN + LSTM	73.5	76.5	74.7
	BCDF	85.6	89.2	88.3

Table 5 shows the recognition performance of different parts of sentences based on the GoEmotions dataset. We can see that BCDF performs the best for identifying the noun, verb, adverb, and adjective words by comparing with the RNN, CNN + RNN, LSTM and CNN + LSTM. For nouns, BCDF achieves 85.6% F1 score. For verbs, BCDF achieves 85.7% F1 score. For adverbs, BCDF achieves 84.8% F1 score. For adjective words, BCDF achieves 85.9% F1 score.

**TABLE 5:** Recognition of different parts of sentence based on GoEmotions dataset (%).

Part of speech	Model	Pre	Rec	F1
Noun	RNN	68.4	73.2	71.5
	CNN + RNN	72.3	76.3	73.8
	LSTM	74.5	76.2	75.5
	CNN + LSTM	73.1	76.7	74.5
	BCDF	84.1	87.1	85.6
Verb	RNN	69.8	74.2	73.3
	CNN + RNN	75.4	77.6	74.7
	LSTM	73.8	78.3	74.6
	CNN + LSTM	75.2	77.7	76.5
	BCDF	82.3	88.3	85.7
Adverb	RNN	64.8	70.2	76.3
	CNN + RNN	69.4	71.6	70.9
	LSTM	73.8	77.3	75.6
	CNN + LSTM	71.2	78.7	75.5
	BCDF	78.1	82.6	84.8
Adjective	RNN	66.8	73.2	70.3
	CNN + RNN	74.4	77.6	75.7
	LSTM	75.8	79.3	74.4
	CNN + LSTM	72.6	75.3	74.8
	BCDF	84.2	87.8	85.9

### Compare Classification Performance of Bi-Direction Convolutional Word Embedding Classification Framework and Long-Term and Short-Term Memory Model

**FIGURE 1:**
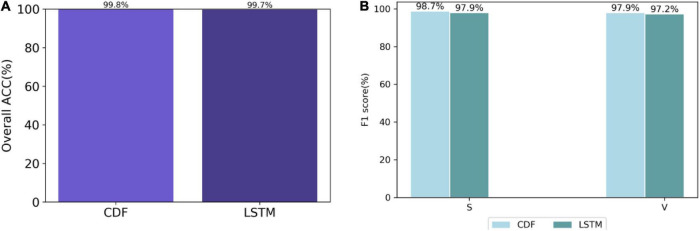
Performance comparison between BCDF and LSTM-based model based on GoEmotions dataset. **(A)** Overall accuracy **(B)** F1 score.

To demonstrate the superiority of the ConvLSTM layer in BCDF, the experiment first compares the BCDF with the LSTM-based model based on the GoEmotions dataset. Figure 1 shows the performance comparison between BCDF and LSTM-based model. As can be seen from Figure 1, the ACC of BCDF is 0.1% higher than that of LSTM-based model. The F1 scores of DCF is 0.8 and 0.5% higher than that of LSTM-based model, respectively.

## Conclusion

This paper proposed a text emotion analysis framework BCDF based on word embedding and splicing. It can express the word vector in the text and embed the part of speech tagging information as a feature of sentence representation. A Bi-LSTM emotional parallel attention mechanism is also presented. As the convolutional layer can better extract certain components of sentences (such as adjectives, adverbs, nouns, etc.), a ConvLSTM network is designed to further improve the classification results. Experimental results showed that the proposed text emotion analysis model has increased 3.3 and 10.9% in terms of F1 score on psychological and news text datasets, respectively. In the future, the time and space efficiency of the proposed BCDF will be improved by using the advanced lightweight convolutional techniques.

## Data Availability Statement

The original contributions presented in this study are included in the article/supplementary material, further inquiries can be directed to the corresponding author.

## Author Contributions

BS proposed the idea, conducted the experiments, and wrote the whole manuscript.

## Conflict of Interest

The author declares that the research was conducted in the absence of any commercial or financial relationships that could be construed as a potential conflict of interest.

## Publisher’s Note

All claims expressed in this article are solely those of the authors and do not necessarily represent those of their affiliated organizations, or those of the publisher, the editors and the reviewers. Any product that may be evaluated in this article, or claim that may be made by its manufacturer, is not guaranteed or endorsed by the publisher.

## References

[B1] Agga A. Abbou A. Labbadi M. Houm Y. E. (2021). Short-term self consumption PV plant power production forecasts based on hybrid CNN-LSTM. ConvLSTM models. *Renew. Energy* 177 101–112. 10.1016/j.renene.2021.05.095

[B2] Angioletti L. Tormen F. Balconi M. (2022). Judgment and Embodied Cognition of Lawyers. Moral Decision-Making and Interoceptive Physiology in the Legal Field. *Front. Psychol.* 13:853342. 10.3389/fpsyg.2022.853342 35401313 PMC8987697

[B3] Chancellor S. Baumer E. P. S. De Choudhury M. (2019). Who is the “human” in human-centered machine learning: the case of predicting mental health from social media. *Proc. ACM Hum. Comput. Interact.* 3 1–32. 10.1145/335924934322658

[B4] Demszky D. Movshovitz-Attias D. Ko J. Cowen A. Nemade G. Ravi S. (2020). GoEmotions: a dataset of fine-grained emotions. *arXiv* [Preprint]. 10.48550/arXiv.2005.00547

[B5] Fukuda N. Granzier H. Ishiwata S. Morimoto S. (2021). Recent Advances on Myocardium Physiology. *Front. Physiol.* 12:697852. 10.3389/fphys.2021.697852 34122154 PMC8189172

[B6] Hamilton R. H. Davison H. K. (2022). Legal and Ethical Challenges for HR in Machine Learning. *Empl. Responsib. Rights J.* 34 19–39. 10.1007/s10672-021-09377-z

[B7] Ji X. Zhao J. Fan L. Li H. Lin P. Zhang P. (2022). Highlighting psychological pain avoidance and decision-making bias as key predictors of suicide attempt in major depressive disorder—A novel investigative approach using machine learning. *J. Clin. Psychol.* 78 671–691. 10.1002/jclp.23246 34542183

[B8] Lai V. Tan V. (2019). “On Human Predictions with Explanations and Predictions of Machine Learning Models: A Case Study on Deception Detection,” in *Proceedings of the Conference on Fairness, Accountability, and Transparency (FAT’19)*, (New York, NY: Association for Computing Machinery), 29–38. 10.1145/3287560.3287590

[B9] Li X. Qu Y. Yin H. (2021). “PalmTree: Learning an Assembly Language Model for Instruction Embedding,” in *CCS’21: The 28th ACM Conference on Computer and Communications Security*, (New York, NY: Association for Computing Machinery), 3236–3251. 10.1145/3460120.3484587

[B10] Linthicum K. P. Schafer K. M. Ribeiro J. D. (2019). Machine learning in suicide science: applications and ethics. *Behav. Sci. Law* 37 214–222. 10.1002/bsl.2392 30609102

[B11] Liu S. Xu J. Feng S. Liao’s Y. (2021). “Framework Design of Anti-online Learning Anomie Behavior System,” in *Advances in Intelligent Information Hiding and Multimedia Signal Processing*, eds Pan J. S. Li J. Tsai P. W. Jain L. C. (Singapore: Springer), 281–288. 10.1007/978-981-33-6420-2_35

[B12] Mehrabi N. Morstatter F. Saxena N. Lerman K. Galstyan A. (2021). A survey on bias and fairness in machine learning. *ACM Comput. Surv.* 54 1–35. 10.1145/3457607

[B13] Mohr D. C. Zhang M. Schueller S. M. (2017). Personal sensing: understanding mental health using ubiquitous sensors and machine learning. *Annu. Rev. Clin. Psychol.* 13 23–47. 10.1146/annurev-clinpsy-032816-044949 28375728 PMC6902121

[B14] Ozawa S. (2021). Emotions induced by recalling memories about interpersonal stress. *Front. Psychol.* 12:618676. 10.3389/fpsyg.2021.618676 33897528 PMC8062919

[B15] Phan L. V. Rauthmann J. F. (2021). Personality computing: new frontiers in personality assessment. *Soc. Personal. Psychol. Compass* 15:e12624.

[B16] Ringeval F. Schuller B. Valstar M. Cummins N. Cowie R. (2019). “AVEC’19: Audio/visual emotion challenge and workshop,” in *Proceedings of the 27th ACM International Conference on Multimedia*, (New York, NY: ACM), 2718–2719. 10.3389/fpsyt.2021.811392

[B17] Sun L. Wang Y. Qu Z. Xiong N. (2021). BeatClass: a Sustainable ECG Classification System in IoT-based eHealth. *IEEE Internet Things J.* 99 1–1.

[B18] Sun L. Zhong Z. Qu Z. Xiong N. (2022). PerAE: an Effective Personalized AutoEncoder for ECG-based Biometric in Augmented Reality System. *IEEE J. Biomed. Health Inform.* [Epub ahead of print]. 10.1109/JBHI.2022.3145999 35077376

[B19] Tan W. Huang P. Li X. Ren G. Chen Y. Yang J. (2021). Analysis of Segmentation of Lung Parenchyma Based on Deep Learning Methods. *J. X-Ray Sci. Technol.* 29 945–959. 10.3233/XST-210956 34487013

[B20] Tate A. E. McCabe R. C. Larsson H. Lundström S. Lichtenstein P. Kuja-Halkola R. (2020). Predicting mental health problems in adolescence using machine learning techniques. *PLoS One* 15:e0230389. 10.1371/journal.pone.0230389 32251439 PMC7135284

[B21] Tung K. (2019). AI, the internet of legal things, and lawyers. *J. Manag. Anal.* 6 390–403.

[B22] Verma S. Dickerson J. Hines K. (2020). Counterfactual explanations for machine learning: a review. *arXiv* [Preprint]. 10.48550/arXiv.2010.10596

[B23] Wang Y. Sun L. Subramani S. C. A. B. (2021). Classifying Arrhythmias based on Imbalanced Sensor Data. *KSII Trans. Internet Inf. Syst.* 15 2304–2320.

[B24] Wang Z. Li T. Xiong N. Pan Y. (2012). A novel dynamic network data replication scheme based on historical access record and proactive deletion. *J. Supercomput.* 62 227–250.

